# Exploring the Potential Impact of a Virtual Body Scan Meditation Exercise Conducted With Pet Dogs on Recipients and Facilitators

**DOI:** 10.3389/fpsyg.2021.698075

**Published:** 2021-07-16

**Authors:** Lori R. Kogan, Cori Bussolari

**Affiliations:** ^1^Department of Clincal Sciences, Colorado State University, Fort Collins, CO, United States; ^2^Department of Clinical Psychology, University of San Francisco, San Francisco, CA, United States

**Keywords:** body scan, virtual, relaxation, anxiety, telehealth, mindfulness, telemental health

## Abstract

Numerous recent studies have shown that COVID-19 and the accompanying mandated lifestyle changes have resulted in significant negative effects on people’s mental health. To meet the increased need for mental health support, while also maintaining physical safety, a variety of telehealth services have been created or expanded. A body scan mindfulness program is an intervention that can easily be modified to be offered virtually. This study was designed to determine if a virtual body scan mindfulness exercise, with participants’ holding their dog or a pillow/blanket, could reduce their stress and anxiety as well as that of the facilitators. Significant differences in pre/post-State Anxiety Assessment scores for participants and facilitators were found. These results are discussed within the framework of the human animal bond and the potential of this form of intervention as a useful virtual tool for participants and facilitators alike.

## Introduction

The 2019 novel coronavirus (COVID-19) has had a devastating global health impact. As of March 25, 2021 hundreds of millions of people have been infected with the virus and millions have died (COVID-19 Map - Johns Hopkins Coronavirus Resource Center, 2021). COVID-19 has created the need for sudden, dramatic lifestyle changes that include physical distancing (often referred to as “social distancing”) and reduced contact with other people (CDC, 2020b). While these steps are seen as vital in the efforts to mitigate the spread of the coronavirus, they have had significant consequences on people’s short- and long-term mental health and wellbeing (Galea et al., 2020).

It was perhaps an example of both understatement and foreshadowing when the Center for Disease Control (CDC) stated in July 2020 that “The coronavirus disease 2019 (COVID-19) pandemic may be stressful for people” (CDC, 2020a). A number of recent studies have shown that indeed, COVID-19 and the accompanying mandated lifestyle changes have resulted in significant negative effects on mental health (Brooks et al., 2020; Pieh et al., 2020a,b; Shigemura et al., 2020). For example, studies conducted in China found higher rates of anxiety, depression, hazardous and harmful alcohol use, and lower mental wellbeing when compared to pre-COVID-19 times (Ahmed et al., 2020; Qiu et al., 2020; Wang et al., 2021). Other countries, including Italy (Rossi et al., 2020), Japan (Ueda et al., 2020), India (Kazmi et al., 2020), Australia (Stanton et al., 2020), and Israel (Levkovich and Shinan-Altman, 2020), have found similar results.

Studies within the United States have mirrored results from other countries (Fitzpatrick et al., 2020). One recent study, for example, reported high rates of anxiety and depression, symptoms of trauma- and stressor-related disorders related to COVID-19, substance use, and suicidality. An alarming 11% of respondents to a recent survey pertaining to mental health reported having seriously considered suicide in the 30 days prior to completing the survey (Czeisler et al., 2020). Elevated rates of suicidality were also found by Ammerman et al. (2021) whereby 18% of survey respondents reported active suicidal ideation in the month prior to completing the survey and 5% reported having attempted suicide in the same period of time (Ammerman et al., 2021). When assessing the impact of demographics on COVID-19 related mental health issues, Zhou (Zhou et al., 2020) found that younger US adults with pre-existing health conditions and less social support reported struggling more with COVID-19-related mental health effects than older, healthy adults with greater support systems. A longitudinal study in the United Kingdom found mental distress highest for women, people between 18 and 34 years of age, and those living with young children (Pierce et al., 2020). Similar results were found in an international meta-analysis which reported high levels of stress, anxiety, and depression as a result of the pandemic in the general population, with people between 21 and 40 years of age, women, and those with higher education levels most impacted (Salari et al., 2020). The negative consequences of these mental health conditions are compounded by the discovery that psychological symptoms and disorders are associated with more severe COVID-19 progression (Yao et al., 2020; Taquet et al., 2021). The results of these studies, documenting the psychological consequences of COVID-19, have resulted in a call for increased efforts and resources to be directed toward intervention and prevention in an effort to help mitigate the negative mental health impact (Zhou et al., 2020).

While the first cases of COVID-19 in the United States were documented in March 2020, the progression of the virus across the world continued throughout the winter holiday season of 2020, adding an additional stressor for many. The holiday season is typically a time when families gather, sharing rituals that make the holidays more enjoyable and promote feelings of family closeness (Páez et al., 2011; Dale et al., 2021). These family rituals play a uniquely important role in holiday enjoyment and are so powerful that even though they are repeated annually, they do not result in typical patterns of satiation; instead appearing to enhance the holiday experience (Sezer et al., 2016). As such, the inability to partake in holiday rituals and gatherings due to COVID-19-related restrictions likely caused additional psychological distress for many. A recent study by Dale et al. in Austria supports this premise, finding significant levels of depression, anxiety, and insomnia over the 2020 holiday season. Furthermore, although the holiday season is a positive time for many, it can also be a stressful time (Páez et al., 2011). For some, the holiday season is accompanied by reduced life satisfaction, mood and emotional wellbeing, and higher suicide rates (Beauchamp et al., 2014; Plöderl et al., 2015; Mutz, 2016; Hofstra et al., 2018). Because it was felt likely that COVID-19 restrictions negatively impacted the 2020 holiday season for many, this study was designed to explore the use of a virtual telehealth stress reduction intervention for US adults during the 2020 holiday season.

### Telehealth Services

Mandated quarantines and social distancing regulations designed in an effort to contain the coronavirus have impacted the ability to deliver psychological services. To help address the increased need for mental health support, while also maintaining physical safety precautions, a variety of telehealth services have been created or expanded (Pereira-Sanchez et al., 2020). Telehealth can be defined as the delivery of psychological and mental health services *via* telecommunication technologies, modalities include telephone-delivered therapy, videoconferencing, mental health apps, and Internet-delivered programs (Nelson et al., 2011).

Research into telehealth in a recent meta-analysis suggests that it is both feasible and effective for treating common mental health disorders (Varker et al., 2019). It has therefore been suggested that telehealth, or more specifically tele-mental health services, is practical, appropriate treatment options during the COVID-19 pandemic and offer several advantages over face to face contact (Chauhan et al., 2020; Zhou et al., 2020). In fact, telehealth is seen by many as an ideal way to support people’s health needs while maintaining the social distancing needed to slow the transmission of the virus (CDC, 2020b; Ramalho et al., 2020; Smith et al., 2020). The increased use of telehealth is also a way to reduce personal protective equipment usage, improve access to care, and reduce the burden on health care systems (Burroughs et al., 2020; Chauhan et al., 2020). A wide array of psychological interventions can be offered remotely. Mindfulness programs are an example of a type of intervention that can easily be modified to be offered virtually both synchronously and asynchronously.

### Mindfulness Programs

Prior to the COVID-19 pandemic, significant numbers of people struggled with stress, anxiety, and depression (Ritchie and Roser, 2018; Kousoulis, 2019; ADAA, 2021), but the pandemic has dramatically increased the need for preventative and supportive psychological interventions (Fitzpatrick et al., 2020). One commonly used supportive intervention is mindfulness-based programs (MBPs). MBPs, used in a variety of settings due to their ease in accessibility, have been found effective in promoting mental health and reducing anxiety, stress, and depression (Call et al., 2014; Colgan et al., 2016; Corbett et al., 2019; Kabat-Zinn, 2019; Galante et al., 2021).

There are several variations of MBPs with some of the most common forms including mindfulness-based stress reduction (MBSR; Kabat-Zinn, 1990), mindfulness-based cognitive therapy (Segal et al., 2018), and body scan meditation (Kropp and Sedlmeier, 2019). The core tenant to all MBP programs is a state of consciousness that is characterized by the self-regulation of attention toward present-moment experiences coupled with an accepting, nonjudgmental stance toward these experiences (Bishop et al., 2004; Kearney et al., 2018). Mindfulness meditation encourages people to practice nonjudgmental awareness of their sensations, emotions, and cognitions and encourages a distancing from one’s internal mental dialog (Klatt et al., 2009). The use of MBSR has grown dramatically with numerous studies, including meta-analyses (Grossman et al., 2004; Chiesa and Serretti, 2009), documenting its efficacy in a variety of medical, social, educational, and workplace settings (Pascoe et al., 2017; Bartlett et al., 2019; Spinelli et al., 2019). It has been found useful for a myriad of symptoms and illnesses including anxiety, depression, eating disorders, chronic pain, and psychological trauma (Kabat-Zinn, 2003; Hilton et al., 2017; Beccia et al., 2018; Taylor et al., 2020). Additionally, virtual reality mindfulness has been recently explored with positive effects noted (Navarro-Haro et al., 2017; Chandrasiri et al., 2020; Seabrook et al., 2020).

The body scan form of mindfulness training is a somatically oriented experience that can be used alone or in combination with MBSR and can last from 5 to 30 min (Kabat-Zinn, 1990; Dreeben et al., 2013). The body scan is an introspective practice in which attention is progressively directed from one part of the body to another, with instructions to simply pay attention to sensations as they arise. The intervention can be delivered *via* a recorded narrative, as was done in the current study. The process encourages several behaviors including turning one’s attention inward and adopting an accepting, nonjudgmental attitude. It invites prolonged, quiet introspection and encourages a better connection with one’s body (Salmon et al., 2011). As such, it promotes somaesthetics, or “body consciousness,” and the notion of the importance of somatic awareness in health and wellness (Shusterman, 1999). Body scan meditation has been found effective in improving self-acceptance, increasing kind behaviors toward oneself, and decreasing negative feelings and self-judgment (Kropp and Sedlmeier, 2019). Additionally, it has been found effective in decreasing cortisol levels (Schultchen et al., 2019) and enhancing cognitive functions, such as reaction time and attention (Adhikari et al., 2018).

While most mindfulness programs involve several sessions spanning numerous weeks (Carmody and Baer, 2009), brief mindfulness interventions offer a viable alternative and can easily be implemented by people in their own home, typically at a lower cost, and offer an example of the self-management model of care (Ussher et al., 2014). Specifically, brief body scan interventions can have immediate positive effects, such as a reduction in cravings and mood-related withdrawal symptoms (Cropley et al., 2007) reduced pain-related distress (Mirams et al., 2013; Ussher et al., 2014), increased attention (Adhikari et al., 2018), and improvement in psychological wellbeing (Sauer-Zavala et al., 2013).

A small number of studies have examined the impact of mindfulness exercises on clinicians. Block-Lerner et al. (2007), for example, found that mindfulness practices may increase therapeutic empathy. Similarly, findings from Horst et al. (2013) suggest that using mindfulness in session may positively impact the therapeutic relationship as it “provided a sense of calm, facilitated conversation, slowed the pace of the session, and was helpful with transitions.” (p. 378). Additionally, Christopher and Maris (2010) suggested that using mindfulness in clinical training programs can enrich the physical and psychological wellbeing of trainees and potentially limit burnout. Despite these positive effects, offering a body scan mindfulness intervention virtually has not yet been evaluated (The Lancet Psychiatry, 2021), and there remains limited research specifically looking at the benefits of a mindfulness intervention for clinicians. This study was designed to determine if a body scan mindfulness exercise, delivered remotely by a facilitator utilizing an audio recording, could reduce the stress and anxiety of those receiving the intervention as well as those delivering it.

Additionally, because of the growing body of the literature noting the positive impact of companion dogs for owners during the COVID-19 pandemic (Carr et al., 2020a; Bussolari et al., 2021; Hoffman, 2021; Johnson and Volsche, 2021), as well as the psychological benefits of dogs (Carr et al., 2020a,b; Gee et al., 2021), this study explored the impact of including participants’ dogs in the body scan mindfulness exercise. The combined research suggesting that mindfulness training as well as companion dogs may both offer positive effects suggest that when combined, may offer a unique option for virtual mindfulness as a tool to reduce stress. It was hypothesized that the inclusion of participants’ dogs in the intervention, when compared to a control group, would enhance the reduction of anxiety and lead to a greater enjoyment of the exercise. Lastly, we hypothesized that facilitators of the mindfulness exercise would enjoy the intervention more and feel more connected to participants who completed the exercise with their dog compared to those who completed the exercise with a pillow or blanket.

## Materials and Methods

Participants were recruited *via* social media (e.g., Facebook and Instagram) and had to be at least 18 years of age, have a companion dog in the home, and be able to participate in a Zoom call with a computer or phone that had audio and visual capabilities. Interested participants were able to sign up by completing a short online (Qualtrics) survey. This survey included detailed information about the study including the presence of a short pre- and post-assessment (less than 5 min) delivered through Qualtrics and the requirement for both audio and visual connection with their facilitator during the exercise. The visual requirement was to ensure that the participants were holding their appropriately assigned item (pillow/blanket or dog). It was also explained that facilitators would be participating in the body scan exercise as well; that they would be doing the exercise together. Additional information on the consent form included their rights as research participants and the condition they had been placed in. Participants were randomly assigned to either a pillow or blanket group, in which they were instructed to hold a favorite pillow or blanket during the body scan exercise or a dog group, in which they were asked to hold or touch their dog during the exercise. After reading the information about the study, people were able to indicate their consent to participate in the research study. This study was reviewed and approved by the Colorado State University Human Subjects Committee (#20-10227H).

To assess the impact of the body scan exercise, participants were asked to complete a short online survey immediately before and after the session. To access the surveys, they were given a URL link to the online surveys created in Qualtrics. To minimize the amount of time the pre/post-surveys would take to complete, the pre-survey was limited to the 20 questions of the State Anxiety Assessment (STAI) and one question asking participants to identify the condition they were in (pillow/blanket or dog). The post-survey was identical to the pre-survey with one additional question asking participants to rate their body scan session on a 5-point scale from 1 – loved it to 5 – hated it. No demographic questions were included in the surveys to minimize the time needed for completion and help ensure one of our goals for the study – namely, to provide a free, easily accessible, anxiety reduction tool to participants, with minimal extra requirements, during a uniquely stressful time.

### Facilitators

It was also hypothesized that the facilitators might benefit from the body scan exercise. A total of 11 facilitators (undergraduate and graduate students from various educational backgrounds) were recruited to participate in the study. To test this theory, facilitators were asked to measure and record their pulse before and after the exercise. If they had a device to measure their pulse, they were allowed to use what they had (e.g., Apple watch). If they did not have such a devise, they were given a finger pulse oximeter (Santamedical Generation 2 Fingertip Pulse Oximeter Oximetry Blood Oxygen Saturation Monitor). Similar to the participants, facilitators were asked to complete an online pre- and post-assessment. These assessments consisted of the 20 STAI questions and a place to note their pulse rate. They were also asked to indicate their enjoyment level of the session (5-point scale from 1 – loved it to 5 – hated it) and how connected they felt to the participant (3-point scale from 1 = very connected, 2 = somewhat connected, and 3 = minimally connected). In addition to the rating scales, facilitators were asked to respond to five open ended questions at the end of the study pertaining to their experiences working on this project.

The STAI (Spielberger, 1983) consists of 40 items, 20 items allocated to the State Anxiety subscale and 20 to the Trait Anxiety subscale. This study only included the 20 items from the State Anxiety Scale (S-Anxiety) subscale. This scale evaluates participants’ current state of anxiety by asking how respondents feel “right now, at this very moment” to a series of items that assess subjective feelings of apprehension, tension, nervousness, and worry. Participants are asked to respond to each question using a 4-point Likert scale with 1 = not at all, 2 = somewhat, 3 = moderately so, and 4 = very much so. Scoring is reverse coded for anxiety-absent items, after which all the items are added together to obtain a total score. Range of scores for the subtest is 20–80, with higher scores indicating greater anxiety. Scores of 39–40 or higher suggest clinically significant symptoms of anxiety (Julian, 2011). Because the STAI was designed to detect transitory states, test-retest coefficients are lower for the State Anxiety than the Trait Anxiety Scale, with scores ranging between 0.31 and 0.86. Internal consistency coefficients for the scale have ranged from 0.86 to 0.95; test-retest reliability coefficients have ranged from 0.65 to 0.75 over a 2-month interval (Spielberger, 1983). The STAI is highly correlated with the Cattell and Scheier’s Anxiety Scale Questionnaire (0.85) and the Taylor Manifest Anxiety Scale (0.73; Spielberger, 1983).

This study is a mixed-method design, reporting both quantitative and qualitative results. Data analyses consisted of split-plot (mixed-design two-way repeated measures) ANOVAs, after testing to ensure all assumptions to conduct an ANOVA were met, to determine any significant difference between conditions (control and dog) on within-subject anxiety level as determined by the State Anxiety Measure (STAI). Because of low numbers of participants within some groups, Fisher’s exact test (Fisher, 1934) was used to assess any differences based on condition group in perception of the experience. Assuming a two-way ANOVA at *α* = 0.05, a sample size of 54 will provide 95% power to detect a difference in change between groups and within participants. Statistical significance was accepted at *p* < 0.05.

### Intervention

The body scan sessions, conducted one-on-one, consisted of facilitators initiating the Zoom call, introducing themselves, and inviting participants to get comfortable and ready to complete the exercise. The participants were reminded what condition they were placed in (pillow/blanket or dog) and asked to have these items or animals in their lap or within touching distance.

Participants were then asked to complete their pre-assessment, while facilitators completed their own pre-assessment. After the assessments were completed, the facilitator began playing the body scan recording, an 8-min guided audio session taped by one of the authors (CB) with extensive experience with counseling and body scan exercises (see appendix for script). Both the participant and the facilitator engaged in the body scan exercise. When it was completed, the facilitators gave participants a moment to reengage with their current surroundings. When ready, the participants were asked to complete the post-assessment by using another URL link copied into the chat feature of the Zoom call. While still in the Zoom meeting, participants and facilitators both completed their post-assessments. This marked the end of the intervention. While participants were completing their assessments, the facilitators completed their own pre- and post-assessments.

A total of 129 participants completed the body scan exercise and the pre- and post-assessments between October 2020 and January 2021. Only those who had a completed pre- and post-assessment were included in the data analysis. Out of the 129 participants, 64 (49.6%) were in the pillow/blanket group and 65 (50.4%) were in the dog group.

### Participants

The pre-assessment STAI scores ranged from 21 to 73 with a mean of 41.45 (*SD* = 11.26), compared to the post-STAI scores that ranged from 20 to 54 with a mean of 30.03 (*SD* = 8.02). A significant difference in pre- and post-STAI scores was found [*F*(1,214) = 260.22, *p* < 0.001, *η*^2^ = 0.55]. No difference was found based on group [dog or control; *F*(1, 214) = 1.65, *p* < 0.201]. There were also no significant differences found in participants’ rating of the sessions based on what condition they were in (*p* = 0.204).

### Facilitators

A group of 11 student facilitators completed 124 pre/post-assessments. A significant difference in pre- and post-STAI scores was found [*F*(1,122) = 164.63, *p* < 0.001, *η*^2^ = 0.574]. No difference was found based on group [dog or control; *F*(1,122) = 0.474, *p* < 0.542]. Pre-score sums ranged from 21 to 63 with a mean of 38.15 (*SD* = 11.03), compared to post-sum scores, ranging from 20 to 53 with a mean of 32.00 (*SD* = 8.71). Pre- and post-pulse rates were also significantly different [*F*(1,122) = 41.506, *p* < 0.001, *η*^2^ = 0.254], with pre-pulse rates ranging from 58 to 107 with a mean of 80.56 (*SD* = 10.14) and post-pulse rates ranging from 55 to 99 with a mean of 77.37 (*SD* = 8.76). No significant differences in pulse based on condition were found [*F*(1,122) = 0.189, *p* = 0.664].

When assessing the facilitators’ rating of the session, there were no significant differences (*p* = 0.27; Table 1). When asked how connected they felt with the participant, there was an association between condition and reported connectivity, with higher feelings of connection to participants in the dog condition when compared to the pillow/blanket condition (Fisher’s exact test, *p* = 0.032, Cramer’s *V* = 0.23). *Post-hoc t*-tests found a significant difference in the number of facilitators who reported feeling minimally connected (*t* = −2.51, *p* = 0.014; Table 2).

**Table 1:** Partcipants’ and facilitators’ rating of the virtual body scan intervention for dog and pillow/blanket groups.

	Loved it		Liked it		It was just OK		Didn’t really like it /hated it		Total	
	Participants	Facilitators	Participants	Facilitators	Participants	Facilitators	Participants	Facilitators	Participants	Facilitators
Pillow/blanket	25 (39.7%)	22 (34.4%)	31 (49.2%)	32 (50.0%)	7 (11.1%)	9 (14.1%)	0	1 (1.6%)	63	64
Dog	36 (55.4%)	23 (38.3%)	24 (36.9%)	34 (56.7%)	5 (7.7%)	3 (5.0%)	0	0	65	60

**Table 2:** Facilitators’ reported connectness with participants for dog and pillow/blanket groups.

Facilitators	Very connected	Somewhat connected	Minimally connected	Total
Pillow/blanket	19 (29.7%)	26 (40.6%)	19 (29.7%)	64
Dog	27 (45.0%)	26 (43.3%)	7 (11.7%)	60
*t*-test	1.77 *p* = 0.079	0.30 *p* = 0.763	−2.51 *p* = 0.014	124

### Facilitators Qualitative Feedback

In addition to the pre- and post-surveys, facilitators were asked to respond to five open-ended questions at the end of the study about their experience. The facilitators expressed positive sentiments regarding their involvement with the virtual body scan intervention, especially during the COVID-19 pandemic. For example, one facilitator noted, “I really enjoyed this experience. The amount of stress that everyone was under at this time was staggering. This was a very timely and needed study.”, while another one similarly commented, “Connecting with different people around the country, meeting their dogs, and helping them relax was incredibly fulfilling. Though COVID-19 was stressful, it felt like we were making the most out of the crazy and hectic time we are living in.”

A few facilitators noted feeling surprised by the effectiveness of the short intervention within a virtual environment. For example, “I was surprised and appreciative of how connected the participants, researchers, and animals could still feel to one another even through video-based platforming.” In addition, another facilitator commented, “I was surprised by how easy it was to connect with people, all over Zoom. I thought it would be difficult and awkward, but it wasn’t.” Interestingly, several facilitators commented upon how much they relished being able to see the participants’ dogs. For instance, when asked to discuss what they enjoyed most about the project, comments, such as “Seeing people hugging and petting their dogs made me feel warm.” and “I really enjoyed ‘meeting’ the participants and their furry counterparts that came from all over…”, were abundant.

Of importance, the facilitators also noted experiencing concurrent feelings of relaxation during the intervention. For example, “I was surprised by how much [exercise] helped me. I felt more relaxed and at ease after each session… and found myself leaning on [the exercise] to help me during times of stress.” Likewise, their experiences subsequently reinforced novel behaviors. For instance, “This relaxation exercise helped me relax as well. I do have a new habit that when I feel tired or stressed, I just play the tape and do the body scan exercise.” During a time when stress was high and social interactions were limited, the ability to virtually spend time supporting people with their dogs was seen as paramount. As one facilitator noted, “Serving as a body scan leader gave me a sense of gratitude. I knew that just taking a small portion of my day to help other people relax would not only benefit the participant but also me. It was so fulfilling to have participants thank me for the experience.”

## Discussion

The results of this study suggest that a body scan exercise, facilitated virtually, can be a viable supportive intervention to reduce anxiety and stress. Of importance, our findings suggest that both participants and facilitators can experience significant decreases in anxiety from completing the exercise. Additionally, facilitators reported feeling more connected to the participants when their dog was present.

These positive outcomes for participants are consistent with previous tele-mental health research (Mikolasek et al., 2018; Sust et al., 2020). This current study was conducted during the 2020 holiday season, a moment in history when society had been plunged into a global pandemic where social isolation was a necessary precaution against potential illness or death. Thus, at a critical time of compounding stressors, access to traditional in-person mental health interventions was not available, creating the need for alternative options. It is therefore notable that one session of virtual body scan meditation helped decrease participants’ anxiety levels, underscoring its economic and practical value.

Equally notable is the findings related to the facilitators’ reduction of anxiety. Although there is a growing body of the literature reporting the benefits (for themselves and the therapeutic relationship) of a personal mindfulness practice for therapists and healthcare providers (Shapiro et al., 2007; Davis and Hayes, 2011; Campbell and Christopher, 2013; Mensah and Anderson, 2015; Dobkin and Lucena, 2016; Hamilton and Marietti, 2017; Bennett-Levy, 2019; Michalak et al., 2020), there is the scant literature on the secondary benefits for those facilitating this practice with others. One study that interviewed clients and therapists about their perceptions of incorporating mindfulness into therapy sessions found several benefits for both including helping both the client and therapist transition into the therapy setting and facilitating feelings of calmness (Horst et al., 2013).

Since the onset of the COVID-19 pandemic, a time that has demanded new ways to deliver therapy since traditional in-person therapy has not been a viable option, mental health professionals have struggled to meet the increased demand for mental health support (Sampaio et al., 2021; Tosone, 2021). Of importance, therapists are tasked with supporting others while concurrently experiencing pandemic related stressors. In light of the increased risk of burnout – which can lead to decreased quality of client care – faced by counselors/therapists during the pandemic, the importance of mental health practitioners addressing their own psychological health is vital (Rupert and Morgan, 2005; Yang and Hayes, 2020; Holmes et al., 2021; Patterson et al., 2021). Facilitators in this study noted that their experience was not only enjoyable and relaxing, but also gave them a sense of purpose through the ability to offer a useful intervention to others. Thus, our findings reflect important ramifications for meeting the increased mental health needs of practitioners during the pandemic.

It is interesting to note that, although not statistically significant, more facilitators and participants indicated loving the experience when participants’ dogs were present during the session compared to sessions in which participants held a pillow or blanket. This finding is consistent with recent human-animal research that has illuminated the comfort and companionship that pets can provide during the COVID-19 pandemic (Bussolari et al., 2021; Holland et al., 2021). These positive effects are perhaps due to pets’ nonjudgmental acceptance and affection (Garrity et al., 1989; Zasloff and Kidd, 1994; Labrecque and Walsh, 2011), and their ability to reduce mental distress and improve their owners’ quality of life (Aydin et al., 2012; Peacock et al., 2012; Hayden-Evans et al., 2018). Similarly, facilitators reported feeling more connected to the participants when dogs were included. It would appear that the dogs were able to serve as social lubricants, even in this virtual setting. Previous research has supported the premise that pets can act as conduits for increased social contact (Guéguen and Ciccotti, 2008; Wells, 2019).

This study has some limitations that should be acknowledged. Firstly, the body scan mindfulness practice was only administered one time per participant, and no data regarding participant demographics, prior experience with mindfulness training, or long-term effects of the intervention were gathered. Further research could address these issues by having participants engage in more than one session and collecting additional data, including a follow-up assessment. Secondly, all the participants were dog guardians, so care should be taken when generalizing to other populations. Because there might be inherent differences between dog guardians and non-dog guardians, it is important for future research to consider including differing species of companion animals as well as participants who do not have a companion animal. It should also be noted that because all facilitators used the same pre-recorded audio file to standardize the procedure and minimize facilitator variability, facilitator effect was not assessed. Future studies might want to limit facilitators to only one session or assess facilitator impact. Lastly, this study was completed during the 2020 holiday season, which, for many, was one of the more stressful times during the COVID-19 pandemic due to increased infection rates and enforced social constraints. It will be important to gather further data to assess the benefits of this short-term telehealth practice under different circumstances.

## Conclusion

In summary, the results suggest that a single virtual body scan mindfulness session can reduce anxiety in both participants and facilitators. The facilitators’ comments indicated that, in addition to the participants, they too reaped the benefits of this intervention. Moreover, the inclusion of a dog appears to increase feelings of connectedness between facilitators and participants.

## Data Availability Statement

The raw data supporting the conclusions of this article will be made available by the authors, without undue reservation.

## Ethics Statement

The studies involving human participants were reviewed and approved by the Colorado State University. Written informed consent for participation was not required for this study in accordance with the national legislation and the institutional requirements.

## Author Contributions

LK and CB contributed to all aspects of this study and approved the submitted version.

### Conflict of Interest

The authors declare that the research was conducted in the absence of any commercial or financial relationships that could be construed as a potential conflict of interest.
